# Study on Status and Willingness towards Hepatitis B Vaccination among Migrant Workers in Chongqing, China: A Cross-Sectional Study

**DOI:** 10.3390/ijerph16204046

**Published:** 2019-10-22

**Authors:** Hui Xiang, Xiaojun Tang, Meng Xiao, Lin Gan, Kun Chu, Shan Li, Yu Tian, Xun Lei

**Affiliations:** 1 School of Public Health and Management, Chongqing Medical University, Chongqing 400016, China; ashely1010@163.com (H.X.); 100108@cqmu.edu.cn (X.T.); felicity_xiao@163.com (M.X.); qq1414154380@163.com (L.G.); chukun@stu.cqmu.edu.cn (K.C.); 2 Research Center for Medicine and Social Development, Chongqing Medical University, Chongqing 400016, China; 3 Innovation Center for Social Risk Governance in Health, Chongqing Medical University, Chongqing 400016, China; 4 School of Clinical Medicine, Chongqing Medical University, Chongqing 400016, China; antonio19960424@icloud.com; 5 Nan’an District Center for Disease Control and Prevention, Chongqing 400067; nacdcty@163.com

**Keywords:** migrant workers, hepatitis B, vaccination status, willingness to vaccinate

## Abstract

Background: Rural-to-urban migrant workers may serve as a bridge population for the cross-regional spread of hepatitis B vaccination (HBV) due to frequent shifts between their work areas and homelands, and they are less likely to be covered by the national hepatitis B (HB) immunization program. This study aimed to investigate the current inoculation status of HB vaccine among migrant workers and the willingness to be vaccinated among non-vaccinated ones. Methods: We conducted a cross-sectional survey using anonymous interviews with migrant workers selected by two-stage cluster sampling from July to December 2018. Binary logistic regression models were adopted to detect influencing factors associated with HB inoculation status and vaccination willingness. Results: 1574 respondents were recruited in the surveys, and 773 (49.11%) respondents reported that they had been inoculated with HB vaccine. Only 285 (35.58%) non-vaccinated respondents were willing to be inoculated. Logistic regression indicated that younger age, higher education level, less wearing of condoms, higher knowledge scores of HB, and higher risk perception of HBV infection were positively associated with inoculation of HB vaccine. Respondents who were more highly educated, and drinkers, with higher knowledge scores of HB and with higher risk perception of HBV infection were more willing to be vaccinated. Conclusions: the HB vaccination rate of migrant workers in Chongqing was relatively low and only a small section of non-vaccinated migrant workers had vaccination willingness. Health interventions and policies are needed to improve knowledge and cognition of HB among migrant workers, particularly for those who are older, less educated, poor in HB knowledge, less likely to wear condoms, and non-drinkers. Peer education, as well as the combination of traditional and new media, would be accessible and effective ways to disseminate HB related knowledge for migrant workers.

## 1. Introduction

Hepatitis B (HB) remains a challenge to public health, causing 500,000 to 750,000 deaths per year due to cirrhosis and liver cancer evolving from HB worldwide [1]. Currently, World Health Organization (WHO) urges countries to invest in eliminating hepatitis once again, and underlined that the low- and middle-countries are still the main battlefields [2]. China is one of the countries with high endemicity of HB reported by the WHO [3], with an estimated number of 93 million HB patients and 100,000 annually new hepatitis B virus (HBV) infections [4]. According to the National Hepatitis Seroepidemiology Survey in 1992 and 2006, the positive rate of hepatitis B surface antigen (HBsAg) of Chinese aged between 1 and 59 decreased from 9.75% to 7.18% [5]. However, the overall HBsAg positive rate was still over 8% for Chinese adults in 2016 [5]. 

HB vaccination has been proved to be the most efficient and economical way to prevent HBV infection and spread [6]. Previous study highlighted that HB vaccine could provide long-term protection against risks of infection encountered in later life [7]. The WHO advocated to enlarge the coverage of HB vaccination prior to 1991 [8], and the Chinese Ministry of Health (MoH) began to offer free HB immunization for neonates after 2002 with free supply being expanded to children under the age of 15 after 2009 [9]. Because of this, the HBsAg positive rate of children under five decreased to 0.32% in 2014 [10], and the reduction of 90% met the WHO’s goals of which the WHO’s Western Pacific Regional Committee had passed a resolution in 2005 to reduce HBV prevalence in children to less than 1% by 2017 [11]. However, China’s adults were not covered by the free immunization program due to the limited health resource [9].A national survey showed that the HBsAg positive rate in populations aged 15 to 59 was 8.75% in 2006 [5].

With rapid development and urbanization, a large number of young adults have migrated from rural areas to big cities for better employment opportunities and higher incomes in China [12]. According to the latest Migrant Workers Monitoring and Investigation Report of China, the number of migrant workers reached up to 288.36 million in 2018 [13]. Migrant workers basically have low education levels and poor self-protected awareness, and most of them live a stressful life and lack of access to health care, where they seem to have a higher likelihood of HBV infection than non-migrant workers [14,15]. The frequent travels make migrant workers a possible bridge population for HBV transmission [16].

A previous study found that the HBsAg positive rates of migrant workers (8.8%) and rural residents (9.1%) were both higher than other groups such as students (7.1%), workers (5.9%), cadres (4.4%), teachers (6.4%), and health care workers (4.9%) in northwestern China [17]. Likewise, HBsAg positive rate of migrant workers (7.05%) is higher than that of local residents (6.32%) in Shenzhen city [18]. The Action Plan for Prevention and Treatment of Viral Hepatitis in China (2017–2020) underlined the need to explore the vaccination strategy and improve HB inoculation for the susceptible and high-risk population [19].

Chongqing is one of the newly promoted first-line metropolises of China where immigration has gradually grown over the years. Nevertheless, studies focused on the HB immunization status among Chongqing’s migrant workers have been rarely seen so far. The present study aimed to investigate the current situation of HB vaccination and vaccinating willingness among migrant workers and detect the potential influencing factors to provide evidence for recommendation and exploration of scale-up immunization programs targeting migrants in China.

## 2. Methods

### 2.1. Study Sites and Participants

The present study was performed in Chongqing, the largest municipality directly under the Chinese central government. Chongqing, located in southwestern China, is referred to as a “miniature of China” because its geographic characteristics, social-economic profile, and urban-rural distribution are close to the national average [20]. The city area of Chongqing, one of the busiest regions for the inflow of China’s migrant population, consists of nine administrative districts with 8.65 million residents, among which immigrants account for 23.5% [21]. In 2007, the estimated HBsAg positive rate was 8.6% among migrant workers in the city area of Chongqing [22]. According to the Health Statistic Yearbook of Chongqing, there were nearly 26,000 new infections of viral hepatitis in 2016 [23].

The target subjects in the study were migrant workers who (1) were 18 years and above, (2) had been in the city area of Chongqing for at least six months, (3) had not registered as Chongqing urban resident, (4) were engaged mainly in the secondary or tertiary industry, such as construction industry, manufacturing industry, wholesale and retail industry, transportation industry, hotel and catering industry, community services. Individuals who were not able to understand the questionnaire items or refused to be surveyed were excluded. 

### 2.2. Sampling Methods

Field surveys were carried out from July to December 2018. Participants were selected by two-stage stratified cluster sampling—first, nine districts of the city area in Chongqing were categorized into three stratifications (very developed, medium developed, and less developed) according to economic background, geographic location and population density, and three districts were then randomly selected from each stratification respectively. Second, two or more enterprise units were purposefully selected within each district, including the manufacturing, construction, wholesale and retail industry, the transportation industry, the hotel and catering industry, community services, with the assistance of local Center for Disease Control and Prevention, Health supervision Institute, and Urban-rural Development Committee. All migrant workers meeting the inclusion criteria were sampled from each enterprise [13].

### 2.3. Study Instruments

The questionnaire was constructed based on the WHO fact sheet on HB immunization and the questionnaires adopted in the published studies on knowledge related to HB epidemics and prevention [24,25,26]. A pilot test was performed with 100 migrant workers in restaurants near to Chongqing Medical University (CQMU) and the framework and wordings of the questionnaire were modified for better understanding of target subjects. The final version consisted of four modules including socio-demographics (16 items), status, and willingness of vaccination (three items), knowledge of HB (14 items), perceived and behavioral risks (eight items). Detailed items and scales of the questionnaire can be found in the Supplementary Materials.

### 2.4. Statistical Analysis

Survey data were double-checked and entered by Epidata 3.1 (The EpiData Association, Odense, Denmark). All data were analyzed using SAS software, version 9.4 (SAS Institute, Cary, North Carolina, USA). Categorical variables were described by number and percentage. Continuous variables were described by the mean and standard deviation (normally distributed variables) or the median and interquartile range (abnormally distributed). Then, univariate analyses were performed using the Chi-square test or Wilcoxon rank sum test to assess the HB vaccination status and willingness of the respondents with variables in the modules of social demographics, knowledge of hepatitis B, and perceived and behavioral risk. Variables with *P*-values less than 0.10 were subsequently inputted into the binary logistic regression model to test the possible influencing factors for two outcomes—(1) HB vaccination status and (2) vaccinating willingness. Adjusted odds ratios (ORs) and 95% confidence intervals (CIs) of variables were computed and the test level was α = 0.05, β = 0.10.

### 2.5. Ethics Statement 

The present study was approved by the Institutional Review Board of CQMU (2018016). Participants were reassured that all responses would be anonymous and written informed consent was secured from each respondent. Peak working hours were avoided to ensure the quality of interviews. 

## 3. Results

A total of 1740 participants met the eligible criteria and 1574 (90.46%) repondents completed this survey. The median age was 32.06 ± 21.18 years, ranging from 18 to 68 while 1487 (94.47%) of respondents were ethnic Han and 87 (5.53%) respondents were ethnic minority. A number of 223 (14.17%) respondents had an education background of primary school or below, and 579 (36.79%) respondents had an education background of junior middle school. 561 (35.64%) respondents had a personal monthly income between 2500 and 4000 RMB, while 364 (23.13%) respondents earned less than 2500 RMB a month (Table 1).

A number of 801 (50.89%) respondents reported that they had not inoculated HB vaccine, among whom there were 516 (64.42%) unwilling to vaccinate. The 745 (42.01%) non-vaccinated respondents were mainly aged between 18 and 30, among whom there were only 285 (35.58%) willing to be vaccinated in the future (Table 2). To explore the reason for not having HB vaccination, 206 (40%) respondents said that they had never heard of HB vaccination, 160 (31.11%) respondents did not know where to vaccinate, 84 (16.30%) respondents doubted the effectiveness or safety of HB vaccine, 55 (10.74%) respondents thought vaccination was inconvenient or inaccessible, and 55 (10.74%) respondents had followed their friends or family members in not vaccinating (Table 3).

The HB knowledge score was 5.00 ± 6.00 and 7.00 ± 3.00 among respondents in non-vaccinated and vaccinated respondents respectively (Table 1). The most popular access for the respondents to obtain HB knowledge and information is hearing from friends or family members (53.88%), followed by television or radio (38.12%), Internet or mobile phone (27.95%), and newspapers or magazine (19.70%) respectively (Table 4). 

A number of 678 (43.07%) respondents thought that it was absolutely impossible/impossible for them to get HB infection. In the last six months, 184 (11.69%) respondents had had casual sex, 92 (5.84%) of them had commercial sex, and 52 (3.30%) respondents had homosexual behaviors or anal sex activities. There were 688 (43.71%) respondents who had had sex with a condom less than five times in the past half a year. In addition, 17 (1.08%) respondents had intravenous drug use by sharing injectors, 23 (1.46%) respondents had experienced illegal blood selling or transfusion, and 298 (18.93%) respondents had shared toothbrush or towels with others in the last six months (Table 1).

All continuous variables were abnormally distributed. As Table 1 shows, univariate analyses using *χ*^2^ test or Wilcoxon sum rank test indicated that HB vaccination status of migrant workers was significantly different with variables of ages, hometown, ethnicity, education background, years of being a migrant worker, job position, working hours per day, monthly personal income, cigarette consumption, knowledge score of HB, perceived risk to HB, commercial sex behavior, and condom use respectively (*P* < 0.05). Binary logistic regression showed that higher education level (OR = 1.85, 95% CI: 1.24–2.74; OR = 1.94, 95% CI: 1.25–3.00; OR = 2.58, 95% CI: 1.57–4.24), higher knowledge scores (OR = 1.17, 95% CI: 1.13–1.22), higher risk perception of HB (OR = 1.40, 95% CI: 1.11–1.75) and less condom usage (OR = 1.35, 95% CI: 1.07–1.70) were associated with having been vaccinated. While age was negatively associated with HB vaccination among migrant workers (OR = 0.98, 95% CI: 0.96–0.99).

As Table 2 shows, univariate analyses using *χ*^2^ test or Wilcoxon sum rank test indicated that the willingness of HB vaccination for non-vaccinated respondents was significantly different with variables of gender, ages, ethnicity, education background, years of being a migrant worker, working hours per day, sending money to family, knowledge score of HB, perceived risk of HB, and condom use (*P* < 0.05). Binary logistic regression showed that migrant workers with higher education level (OR = 2.01, 95% CI: 1.24–3.28; OR = 2.21, 95% CI: 1.26–3.89), drinking (OR = 1.84, 95% CI: 1.23–2.76), with better knowledge scores (OR = 1.13, 95% CI: 1.08–1.19) and higher risk perceptions of HB (OR = 2.18, 95% CI:1.57–3.03) were more willing to be inoculated. Respondents of ethnic minority were unwilling to vaccinate compared with those of ethnic Han (OR = 0.46, 95% CI: 0.22–0.95). 

## 4. Discussion

According to the political and economic context, HB vaccination rates vary across different locations in China. In the present study, 49.11% of migrant workers in the city area of Chongqing had been inoculated with HB vaccine, which was higher than those of migrant workers in Beijing (37.05%), Hebei province (23.58%), Heilongjiang province (21.05%), Jiangsu province (21.75%), Ningxia province (32.81%), and Hainan province (17.09%), but lower than Shenzhen (70.21%) [9,18]. In addition, the amount of non-vaccinated migrant workers, as much as 51.89% of respondents, was very much close to the vaccination rate (51.60%) reported by Gong et al. in 2006 [22], and 42.01% of non-vaccination respondents were mainly aged between 18 and 30 in our study. This suggested that in the past decades the implementation of the HB immunization program had been inefficient in the countryside of Chongqing and surrounding area, from where the migrant workers mainly flow into city area of Chongqing. Therefore, more specific and stronger policies and regulations are needed to enhance HB immunization in rural areas where the migrant workers mostly come from.

A negative association was detected between HB vaccination status and age, which is consistent with the study of Liu et al. [9]. On the one hand, aged respondents in our study might be beyond the required age for free HB immunization by the MoH. On the other hand, aged respondents might take more care of the health of children than themselves [6,9]. In accordance with the study of Yan et al., our findings showed that the more highly educated respondents had a higher vaccination rate and were more willing to be vaccinated if they had not been vaccinated before [27]. Evidence showed that good educational background leads to good cognition of disease prevention among adults, of which the vaccination status of migrant workers differs over educational deviations [28]. In addition, health education campaigns, carried out by staff associations and health service organizations, are also needed to improve migrant workers’ attitude both at the workplace and in the community where they live. 

HB knowledge scores were shown to be positively associated with both vaccination status and willingness of respondents, because good understanding of HB related knowledge probably results in concern of HBV infection and awareness of self-protection towards HB [9]. With regard to the ways of obtaining knowledge, friends or family members were the favorite source for migrant workers, which suggests that health interventions based on peer education might work to a large extent. Moreover, TV, newspapers, and magazines were still popular among respondents, and Internet/mobile phones also played an important role in information updates for migrant workers, which indicates that health education could be promoted by a combination of traditional and new media.

Consistent with the study by Xie et al., Ethnic Han was detected to be positively associated with willingness to vaccinate compared with the ethnic minorities [29]. Religious faith and briefs have been mentioned as influencing factors of immunization behaviors in previous studies [30], which could be an interpretation for the significantl difference in HB vaccination between migrant workers of ethnic Han and other minorities from nearby provinces like Yunnan or Guizhou. 

Consistent with previous study, the perceived risk of HBV infection was positively associated with HB vaccination [9]. It makes sense that the more susceptible respondents perceive the risk of infection with HBV the more active they are to vaccinate [31]. Less condom use in the last six months was negatively associated with HB vaccination. A possible reason was that the vaccinated respondents actually dare to have unprotected sex [32]. Respondents who were alcohol consumers had a strong HB vaccination willingness. It is generally accepted that long-term drinking might be a potential cause of liver damage. Therefore, drinkers are concerned more about liver protection and are more willing to be inoculated. 

Unlike some published studies, income was not detected to significantly affect either HB vaccination or vaccinating willingness as the balanced distributions within every income levels in our study showed [33]. Having said that, 8.15% of non-vaccinated respondents argued that too much self-payment was a big obstacle. Therefore, more subsidy or reimbursement targeting non-vaccinated adults is necessary to explore to reduce the financial burden of migrant workers [9].

In addition, only 35.58% of non-vaccinated respondents expressed willingness to vaccinate in the future. The top-three reasons for their missing of HB vaccination were not having heard of the vaccination, unclear where the vaccination sites were situated, and distrustful of the effectiveness or safety, which suggested that an urgent task of enhancing publicity of HB knowledge and dissemination of health and immunization services should be advanced not only by health education and promotion but also by joint effort and cooperation among the relevant administrative departments.

## 5. Limitations of the Study

Some limitations of the present study should be acknowledged. First, as it was a cross-sectional study, any causal inference should be made cautiously on the basis of the association observed in our study. Second, respondent non-random sampling was adopted due to the limitation of the availability and acceptance of subjects, which may cause a selection bias in the occupational distribution between sampled respondents and the overall migrant workers in Chongqing. Third, data collected by interviews may generate a recall bias both on HB vaccination status and risk behaviors. Therefore, a longitudinal study with a larger sample size by quota sampling, matching with vaccination records or serological examination, is needed to make the findings more robust. 

## 6. Conclusions

Approximately one half of the migrant workers had been inoculated with HB vaccine in the present study. The non-vaccinated respondents were relatively low in their willingness to be vaccinated. Younger age, higher education levels, higher knowledge scores, higher perceived risk of HB, and less condom use were associated with HB vaccination for migrant workers. Migrant workers who were better educated, drank alcohol, with higher knowledge scores and higher perception of HB susceptibility were more willing to inoculate HB vaccine. Health education and campaigns related to HB prevention should be focused on non-vaccinated migrant workers who are older, of lower education, and poor in HB knowledge. Peer education works for the circulation of HB knowledge, and new social media like the internet and smart phone apps will play an important role in addition to traditional social media like television, radio, and magazines.

## Figures and Tables

**Table 1:** Univariate analysis and binary logistic regression on factors associated with vaccination status in hepatitis B vaccination (HBV) vaccine for migrant workers (N = 1574).

Variables	Total	Univariate Analysis				Binary Logistic Regression	
		Non-Vaccinated (N = 801)	Vaccinated (N = 773)	χ^2^/Z ^a^	*P* ^b^	OR ^c^	95% CI ^d^
**Gender**							
Male	781	412 (52.75)	369 (47.25)	2.15	0.142		
Female	793	389 (49.05)	404 (50.95)				
**Age**		39.42 ± 23.94	29.53 ± 14.85	−8.50	<0.001	0.98	(0.96, 0.99)
**Hometown**							
Rural area in Chongqing	1121	593 (52.90)	528 (47.10)	6.29	0.012	Ref	
Rural area in other cities/provinces	453	208 (45.92)	245 (54.08)			1.19	(0.94, 1.52)
**Ethnicity**							
Han	1487	747 (50.24)	740 (49.76)	4.61	0.032	Ref	
Minority	87	54 (62.07)	33 (37.93)			0.66	(0.40, 1.08)
**Education background**							
Primary school or below	223	173 (77.58)	50 (22.42)	112.99	<0.001	Ref	
Junior middle school	579	318 (54.92)	261 (45.08)			1.85	(1.24, 2.74)
High school	524	231 (44.08)	293 (55.92)			1.94	(1.25, 3.00)
College and above	248	79 (31.85)	169 (68.15)			2.58	(1.57, 4.24)
**Marital status**							
Single	367	182 (49.59)	185 (50.41)	1.74	0.419		
Married/Having a relationship	1142	581 (50.88)	561 (49.12)				
Divorced/Widowed	65	38 (58.46)	27 (41.54)				
**Live with the spouse/partner**							
No	778	391 (50.26)	387 (49.74)	0.25	0.620		
Yes	796	410 (51.51)	386 (48.49)				
**Accommodation**							
Self-renting room/Self-purchased house	861	429 (49.83)	432 (50.17)	0.86	0.354		
Co-renting room/Dormitory	713	372 (52.17)	341 (47.83)				
**Years of being a migrant worker**		13.00 ± 10.92	5.00 ± 8.00	−4.55	<0.0001	0.98	(0.97, 1.00)
**Type of work**							
Secondary industry ^e^	851	428 (50.29)	423 (49.71)	0.26	0.608		
Tertiary Industry ^f^	723	373 (51.59)	350 (48.41)				
**Job Position**							
Ordinary employee	1293	694 (53.67)	599 (46.33)	22.46	<0.001	Ref	
Team/Administrator	281	107 (38.08)	174 (61.92)			1.07	(0.82, 1.40)
**Working hours per day**		9.00 ± 4.00	10.00 ± 4.00	2.85	0.004	1.04	(0.98, 1.10)
**Monthly personal income (RMB)**							
<2500	364	194 (53.30)	170 (46.70)	6.19	0.045	Ref	
2501~4000	561	301 (53.65)	260 (46.35)			0.79	(0.57, 1.09)
>4000	649	306 (47.15)	343 (52.85)			1.07	(0.75, 1.53)
**Do you regularly send money to your family?**							
No	440	217 (49.32)	223 (50.68)	0.60	0.437		
Yes	1134	584 (51.50)	550 (48.50)				
**Cigarette consumption**							
No	1131	550 (48.63)	581 (51.37)	8.21	0.004	Ref	
Yes	443	251 (56.66)	192 (43.34)			0.96	(0.74, 1.24)
**Alcohol consumption**							
No	1208	613 (50.75)	595 (49.25)	0.04	0.835		
Yes	366	188 (51.37)	178 (48.63)				
**Knowledge score of HB**		5.00 ± 6.00	7.00 ± 3.00	11.25	<0.001	1.17	(1.13,1.22)
**Do you think you are susceptible to HB?**							
Absolutely impossible/Impossible	678	362 (53.39)	316 (46.61)	9.17	0.010	Ref	
Neutral	121	72 (59.50)	49 (40.50)			1.04	(0.68, 1.60)
Absolutely possible/Possible	775	367 (47.35)	408 (52.65)			1.40	(1.11, 1.75)
**Did you have casual sex in the last six months?**							
No	1390	698 (50.22)	692 (49.78)	2.16	0.142		
Yes	184	103 (55.98)	81 (44.02)				
**Did you have commercial sex in the last six months?**							
No	1482	745 (50.27)	737 (49.73)	3.89	0.049	Ref	
Yes	92	56 (60.87)	36 (39.13)			0.86	(0.53, 1.40)
**Did you have homosexual behavior or anal sex activities in the last six months?**							
No	1522	772 (50.72)	750 (49.28)	0.51	0.474		
Yes	52	29 (55.77)	23 (44.23)				
**Did you use condom less than five times when you had the above sex behavior in the past six months?**							
No	886	500 (56.43)	386 (43.57)	24.93	<0.001	Ref	
Yes	688	301 (43.75)	387 (56.25)			1.35	(1.07, 1.70)
**Did you share an injector for intravenous drug use in the last six months?**							
No	1557	793 (50.93)	764 (49.07)	0.10	0.751		
Yes	17	8 (47.06)	9 (52.94)				
**Did you sell blood or have blood transfusions at illegal clinics in the last six months?**							
No	1551	790 (50.93)	761 (49.07)	0.09	0.767		
Yes	23	11 (47.83)	12 (52.17)				
**Did you share toothbrushes/towels in the last six months?**							
No	1276	649 (50.86)	627 (49.14)	0.002	0.964		
Yes	298	152 (51.01)	146 (48.99)				

^a^ Chi-square test or Wilcoxon sum rank test; ^b^ *P*-value; ^c^ Odds ratio; ^d^ 95% confidence interval; ^e^ Secondary industry includes manufacturing industry and construction industry; ^f^ Tertiary industry includes wholesale and retail industry, transportation industry, hotel and catering industry, community services.

**Table 2:** Univariate analysis and binary logistic regression analysis on factors associated with vaccinate willingness in HBV vaccine for migrant workers (N = 801).

Variables	Total	Univariate Analysis ^a^				Binary Logistic Regression	
		Unwilling to Vaccinate (N = 516)	Willing to Vaccinate (N = 285)	χ^2^/Z	*P*	OR ^b^	95% CI
**Gender**							
Male	412	280 (67.96)	132 (32.04)	4.64	0.031	Ref	
Female	389	236 (60.67)	153 (39.33)			1.18	(0.82, 1.69)
**Age**		40.66 ± 24.85	32.58 ± 21.24	−4.54	<0.001	0.99	(0.97, 1.01)
**Hometown**							
Rural area in Chongqing	593	387 (65.26)	206 (34.74)	0.71	0.401		
Rural area in other cities/provinces	208	129 (62.02)	79 (37.98)				
**Ethnicity**							
Han	747	473 (63.32)	274 (36.68)	5.84	0.016	Ref	
Minority	54	43 (79.63)	11 (20.37)			0.46	(0.22, 0.95)
**Education background**							
Primary school or below	173	139 (80.35)	34 (19.65)	27.86	<0.001	Ref	
Junior middle school	318	202 (63.52)	116 (36.48)			2.01	(1.24, 3.28)
High school	231	130 (56.28)	101 (43.72)			2.21	(1.26, 3.89)
College and above	79	45 (56.96)	34 (43.04)			1.98	(0.97, 4.05)
**Marital status**							
Single	182	113 (62.09)	69 (37.91)	0.63	0.731		
Married/Having a relationship	581	379 (65.23)	202 (34.77)				
Divorced/Widowed	38	24 (63.16)	14 (36.84)				
**Live with spouse/partner**							
No	391	241 (61.64)	150 (38.36)	2.58	0.108		
Yes	410	275 (67.07)	135 (32.93)				
**Accommodation**							
Self-renting room/Self-purchased house	429	367 (62.24)	162 (37.76)	1.92	0.166		
Co-renting room/Dormitory	372	249 (66.94)	123 (33.06)				
**Years of being a migrant worker**		7.09 ± 12.00	6.00 ± 8.00	−2.61	0.009	0.97	(0.94, 1.02)
**Type of work**							
Secondary industry ^c^	428	282 (65.89)	146 (34.11)	0.86	0.353		
Tertiary Industry ^d^	373	234 (62.73)	139 (37.27)				
**Job Position**							
Ordinary employee	694	454 (65.42)	240 (34.58)	2.26	0.133		
Team/Administrator	107	62 (57.94)	45 (42.06)				
**Working hours per day**		9.00 ± 3.00	10.00 ± 4.00	2.41	0.016	1.07	(0.98, 1.17)
**Monthly personal income (RMB)**							
<2500	194	132 (68.04)	62 (31.96)	1.63	0.442		
2501–4000	301	188 (62.46)	113 (37.54)				
>4000	306	196 (64.05)	110 (35.95)				
**Do you regularly send money to your family?**							
No	217	364 (62.33)	220 (37.67)	4.11	0.043	Ref	
Yes	584	152 (70.05)	65 (29.95)			0.66	(0.45, 1.02)
**Cigarette consumption**							
No	550	346 (62.91)	204 (37.09)	1.75	0.186		
Yes	251	170 (67.73)	81 (32.27)				
**Alcohol consumption**							
No	613	406 (66.23)	207 (33.77)	3.74	0.053	Ref	
Yes	188	110 (58.51)	78 (41.49)			1.84	(1.23, 2.76)
**Knowledge score of HB**		4.00 ± 5.00	6.00 ± 5.00	5.45	<0.001	1.13	(1.08, 1.19)
**Do you think you are susceptible to HB?**							
Absolutely impossible/Impossible	362	256 (70.72)	106 (29.28)	28.53	<0.001	Ref	
Neutral	72	58 (80.56)	14 (19.44)			0.64	(0.33, 1.24)
Absolutely possible/Possible	367	202 (55.04)	165 (44.96)			2.18	(1.57, 3.03)
**Did you have casual sex in the last six months?**							
No	698	451 (64.61)	247 (35.39)	0.09	0.766		
Yes	103	65 (63.11)	38 (36.89)				
**Did you have commercial sex in the last six months?**							
No	745	479 (64.30)	266 (35.70)	0.07	0.789		
Yes	56	37 (66.07)	19 (33.93)				
**Did you have homosexual behavior or anal sex activities in the last six months?**							
No	772	496 (64.25)	276 (35.75)	0.27	0.603		
Yes	29	20 (68.97)	9 (31.03)				
**Did you use a condom less than five times when you had the above sex behavior in the past six months?**							
No	500	343 (68.60)	157 (31.40)	10.14	0.001	Ref	
Yes	301	173 (57.48)	128 (42.52)			1.24	(0.89, 1.72)
**Did you share an injector for intravenous drug use in the last six months?**							
No	793	509 (64.19)	284 (35.81)	1.88	0.271 ^e^		
Yes	8	7 (87.50)	1 (12.50)				
**Did you sell blood or have blood transfusions at illegal clinics in the last six months?**							
No	790	507 (64.18)	283 (35.82)	1.47	0.344		
Yes	11	9 (81.82)	2 (18.18)				
**Did you share toothbrushes/towels in the last six months?**							
No	649	426 (65.64)	223 (34.36)	2.22	0.136		
Yes	152	90 (59.21)	62 (40.79)				

^a^ Chi-square test or Wilcoxon sum rank test; ^b^ Odds ratio; ^c^ Secondary industry includes manufacturing industry and construction industry; ^d^ Tertiary industry includes wholesale and retail industry, transportation industry, hotel and catering industry, community services; ^e^ Fisher Exact Test. CI: confidence interval.

**Table 3:** The reasons for non-vaccination (N = 516).

Non-Vaccination Reasons	N (%)
Never heard of HB vaccination	206 (40.00%)
Do not know where to vaccinate	160 (31.11%)
Distrust vaccine’s effectiveness or safety	84 (16.30%)
Inconvenient to get access	55 (10.74%)
Friends/family members have not vaccinated	55 (10.74%)
Too much self-payment required	42 (8.15%)
Have been infected with HBV	12 (2.22%)

**Table 4:** Access to hepatitis B (HB) knowledge (N = 1299).

Source of Obtaining HB Knowledge	N (%)
Friends or family members	848 (53.88%)
Television or radio	600 (38.12%)
Internet or mobile phone	440 (27.95%)
Newspapers or magazines	310 (19.70%)
Doctors	304 (19.31%)
Brochure or booklets	296 (18.81%)
Advertisement	172 (10.93%)
Health education or professional training	133 (8.45%)
Other	115 (7.31%)

## Supplementary Materials

The following are available online at https://www.mdpi.com/1660-4601/16/20/4046/s1, Questionnaire for migrant workers.
