# Supplementary material for: Study on Status and Willingness towards Hepatitis B Vaccination among Migrant Workers in Chongqing, China: A Cross-Sectional Study
Source: Int J Environ Res Public Health. 2019 Oct 22;16(20):4046. doi: 10.3390/ijerph16204046 (PMC6843977; doi:10.3390/ijerph16204046)
Supplement: Supplementary file 1 [file ijerph-16-04046-s001.pdf]

Supplementary materials

Questionnaire for migrant workers

Questionnaire number__________ Industry __________Investigator___________

Hello! We are investigators from Chongqing Medical University. This survey aims to know your understanding of and behaviors related to hepatitis B. It may take 15-20 minutes for you to complete the questionnaire. **The survey is anonymous.** Your information collected by the survey is only for the use of scientific research by which the government will improve the health policy education strategy. Please answer each item according to your actual situation. We are very appreciated for your participation and cooperation!

Please circle the option that match your actual situation or fill out the blank.

Examples :4.Ethnicity: ①Han ②others

PART ONE: Social demographics

1.Gender: ①male②female

2.Date of birth:

3.Hometown: province: district

4.Ethnicity: ①Han②others

5.Education background:

①primary school or below②junior middle school ③senior middle school ④ college /university or above

6.Marital status:

①single ②having a relationship but unmarried ③married ④divorced or widowed

7.Are you living with your spouse/lover at present?

①yes ②no

8.Accommodation:

①renting a room by yourself ②co-renting apartment ③dormitory ④self-purchased house ⑤live with family members

9.How many years have you been a migrant worker: years（months）

10.Current job type:

① manufactory industry ② construction industry ③ wholesale and retail trade ④ transportation, warehousing and postal services ⑤accommodation and catering industry⑥ resident services, repairs and other services ⑦others________(to be specific)

11.Current job position:

①general employee ②team leader ③administrator

12.Working hours per day: hours

13.Monthly personal income (RMB):

①≤1000②1001~2500 ③2501~4000 ④4001~6000 ⑤≥6001

14.Do you regularly send money to your family?

①yes, I send family to RMB per month ②no

15.Do you smoke?

①no ②yes ③have already quit

16.Do you drink?

①no ②yes ③have already quit

PART TWO Status and willingness of vaccination

17.Have you ever been inoculated HB vaccine before?

①yes（**go to question 20**）②no ③I don′t know

18.Would you like to be inoculated HB vaccine at present (self-paying)?

①much unwilling②unwilling③neutral ④willing ⑤strongly willing

19.Why haven′t you inoculated HB vaccine before? (**multiple-choice question**)

①never heard of HB vaccination ②did not know where to vaccinate ③inconvenient to get access to vaccination④distrust vaccine′s effectiveness or safety ⑤too expensive by self-paying ⑥friends/family members have not vaccinated ⑦have infected with HBV⑧ others________(to be specific)

PART THREE Knowledge of hepatitis B

20.Is a hepatitis B virus (HBV) carrier the "hepatitis B patient"?

①yes ②no ③I don′t know

21.Can HBV carriers be identified by their looks?

①yes ②no ③I don′t know

22.Will HBV be infected through damaged skin?

①yes ②no ③I don′t know

23.Will HBV be infected through daily contact?

①yes ②no ③I don′t know

24.Will a newborn baby be infected with HBV by his/her mother?

①yes②no③I don′t know

25.Will a baby be infected with HBV through breast-feed?

①yes ②no ③I don′t know

26.Is it possible to infect with HBV when having sex without condoms?

①yes②no③I don't know

27.Is it possible to infect with HBV by sharing the injection syringes for taking drugs?

①yes ②no ③I don't know

28.Is it possible to infect with HBV when working with HBV carriers?

①yes ②no ③I don't know

29.Is it possible to infect with HBV when living with HBV carriers?

①yes ②no ③I don't know

30.Is HB vaccine able to prevent HBV infection?

①yes ②no ③I don't know

31.Do you think a hepatitis B patient could be completely cured?

①yes ②no③I don't know

32.Do you know the HB surface antigen test?

①yes ②no

33.By which way did you learn about HB-related knowledge as follows? **(multiple-choice question**）

①TV and radio ②newspaper and magazine ③internet ④family members/ friends ⑤doctor⑥practitioner training⑦leaflet/booklet ⑧health lecture ⑨outdoor advertisement ⑩others(to be specific) ⑾I don't know

PART FOUR Perceived and behavioral risk

34.Do you think you are susceptible to HB?

①not susceptible②little susceptible③neutral④susceptible⑤very susceptible

35.How often did you have casual sex in the last six months? (**commercial sex behavior was not be included**)

①never ②rarely（less than five times）③seldom（once or twice a month）④sometimes（once or twice a week）⑤often（more than three times a week）

36. How often did you have commercial sex behavior in the last six months?

①never ②rarely（less than five times）③seldom（once or twice a month）④sometimes（once or twice a week）⑤often（more than three times a week）

37. How often did you have homosexual behavior with men or anal sex behavior in the last six months？

①never ②rarely（less than five times）③seldom（once or twice a month）④sometimes

（once or twice a week）⑤often（more than three times a week）

38.Why did you wear a condom less than five times when you had above sex behaviors in the last six months? （**multiple-choice question**）(**Participants who have no above behaviors or wear a condom more than five times in the last six months could skip the question.**)

①uncomfortable when wearing a condom ②embarrassing if purchase condom ③trust each other ④sex partners did not require to use it ⑤forget to use ⑥do not know how to use condom ⑦have other methods of contraception ⑧too expensive ⑨others(to be specific)

39. How often did you have been injected drugs in the last six months?

①never(go to question 41)②rarely（less than five times）③seldom（once or twice a month）④sometimes（once or twice a week）⑤often（more than three times a week）

40.Did you share the injector with others when you injected drugs?

①never②rarely（less than five times）③seldom（once or twice a month）④sometimes（once or twice a week）⑤often（more than three times a week）

41.How often did you sell/transfuse blood illegally in the last six months?

①never②rarely（less than five times）③seldom（once or twice a month）④sometimes（once or twice a week）⑤often（more than three times a week）

42. How often did you share towels or toothbrushes with others in the last six months?

①never②rarely（less than five times）③seldom（once or twice a month）

④sometimes（once or twice a week）⑤often（more than three times a week）

Survey date________ Investigator________ Verifier________
